# Supplementary material for: Human epidermal Langerhans cells induce tolerance and hamper T cell function upon tick-borne pathogen transmission
Source: Nat Commun. 2025 Nov 28;16:11715. doi: 10.1038/s41467-025-66821-6 (PMC12753675; doi:10.1038/s41467-025-66821-6)
Supplement: Supplementary file 1 — Supplementary Information [file 41467_2025_66821_MOESM1_ESM.pdf]

Supplementary Information

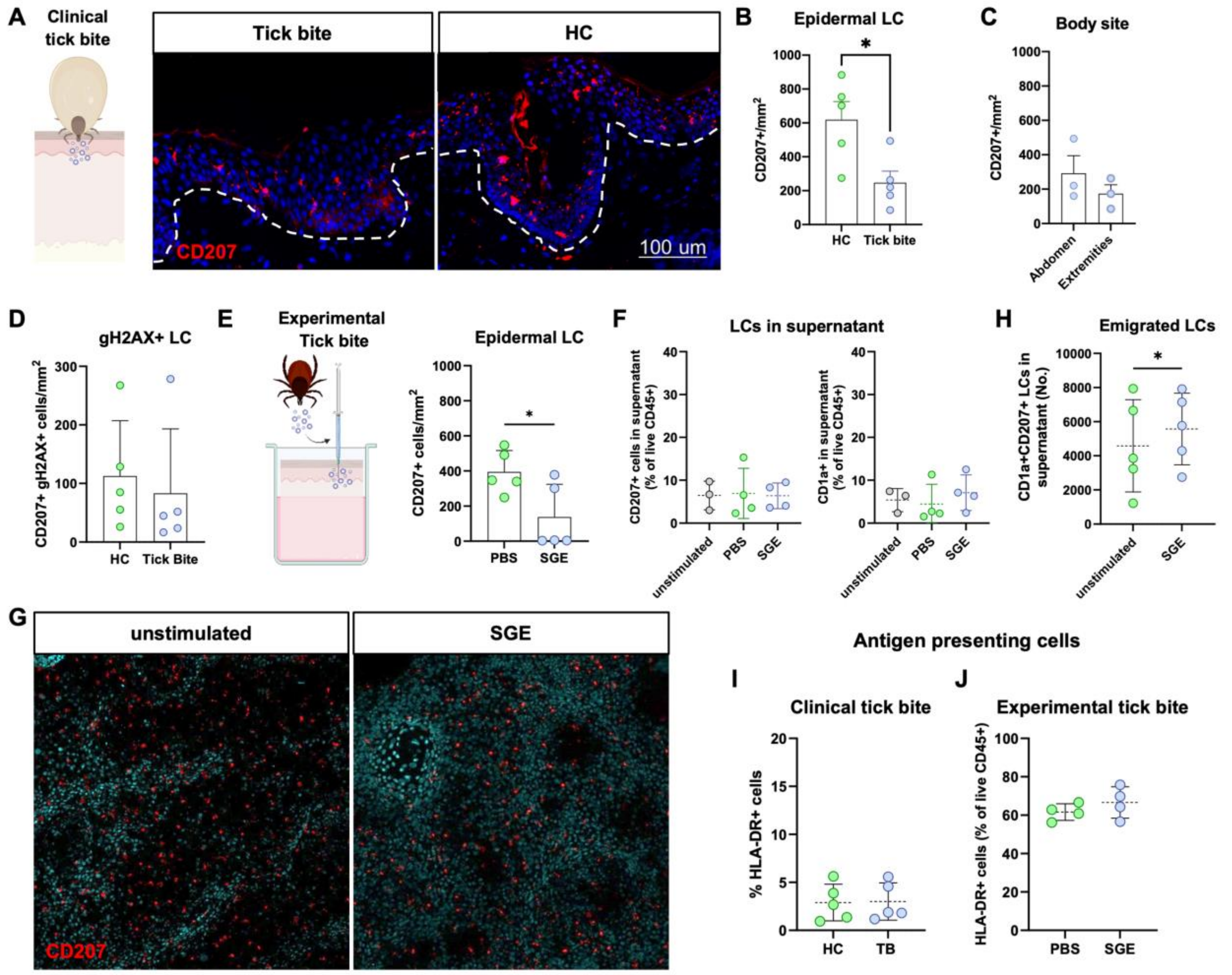

Supplementary Figure 1. Decreased numbers of LCs in clinical and experimental tick bites. (A) Representative IF images of sections from a clinical tick bite compared to healthy control (HC) skin labeled with CD207 (red) and DAPI (blue). Graphic created in BioRender. Stary, G. (2025) https://BioRender.com/o1cvxpj. (B) Quantification of absolute numbers of epidermal CD207+ LCs in clinical tick bite skin biopsies (n=5, mean +/- SD). (C) Distribution of LC numbers in the skin at different body sites in clinical tick bite biopsies (n=6). (D) Quantification of absolute numbers of gH2AX+ LCs in clinical tick bite and HC skin biopsies (n=5, mean +/- SD). (E) Quantification of absolute numbers of epidermal CD207+ LCs in experimental tick bite skin biopsies (n=5, mean +/- SD). (F) Percentages of LCs defined as CD45+CD207+ cells (left panel) or CD45+CD1a+ cells (right panel) migrated to the supernatant after injection of PBS or SGE in the experimental *ex vivo* tick bite model as measured by flow cytometry (n=4, +/- SD). (G) IF staining of CD207+ (red) LCs in epidermal sheets incubated with SGE or media. Nuclei were counterstained with DAPI (cyan).

(H) Quantification of LCs emigrated from epidermal explants (G) into the supernatant after injection of SGE (n=5, mean +/-SD) (I) Numbers of HLA-DR+ cells in clinical tick bite and HC skin biopsies (n=5, mean +/-SD), measured by tissue immunofluorescence. (J) Percentages of HLA-DR+ cells among CD45+ live cells after injection of PBS or SGE in the experimental *ex vivo* tick bite model as measured by flow cytometry (n=4, mean +/-SD). Statistical testing was performed using paired and unpaired Student's *t* test (two tailed, *P < 0.05).

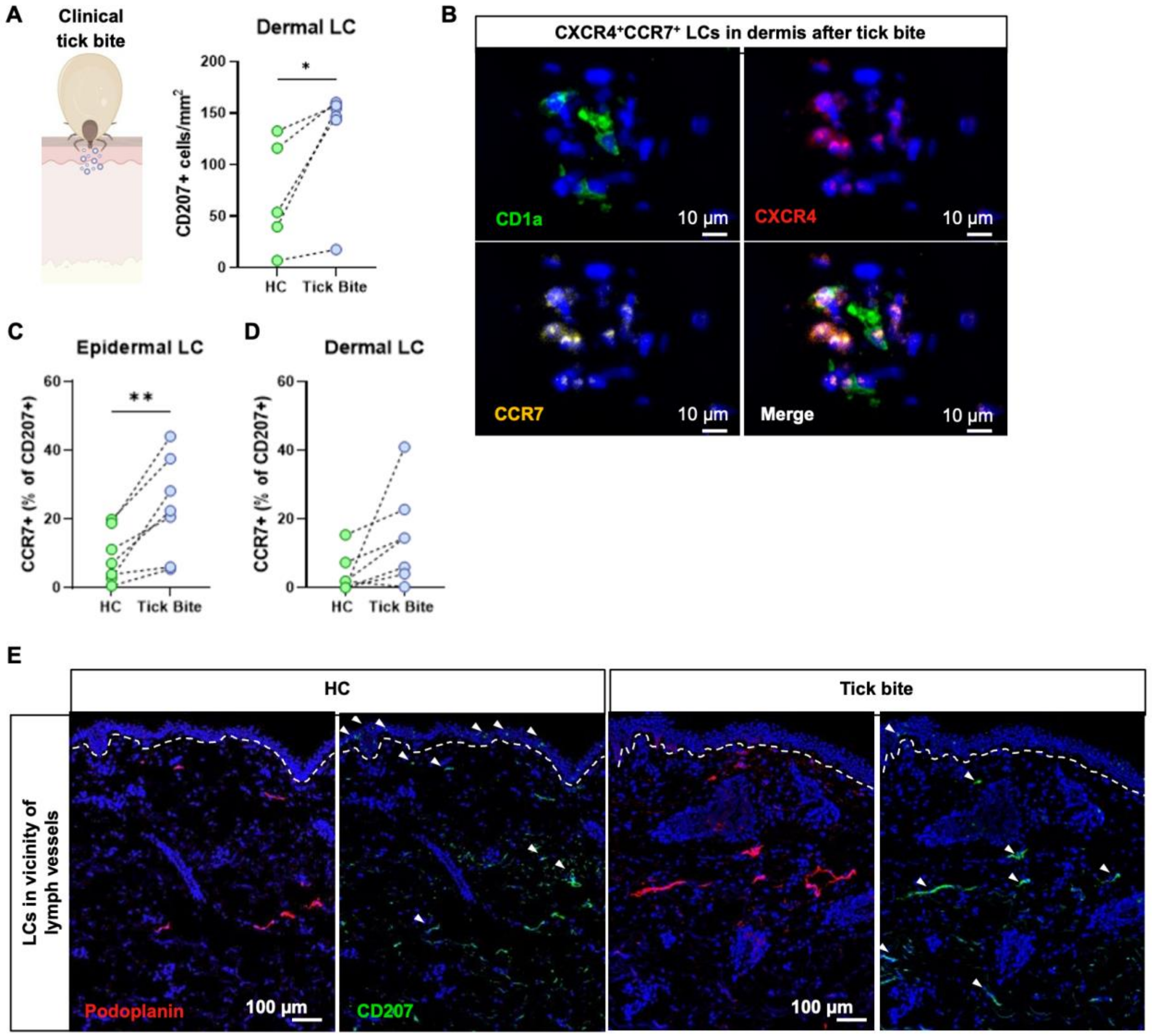

Supplementary Figure 2. LCs express lymph node homing markers and migrate to the dermis upon tick feeding. (A) Quantification of CD207⁺ dermal LCs in clinical tick bite skin biopsies (n=5). Graphic created in BioRender. Stary, G. (2025) https://BioRender.com/o1cvxpj. (B) Representative immunofluorescence images of CXCR4 (red) and CCR7 (yellow) expressing dermal LC. (C-D) Percentage of CCR7 expressing epidermal (C) and dermal (D) CD207⁺

LCs (n=7). (E) Representative immunofluorescence images of the distribution of CD207 (green) expressing LCs and lymph vessels (red) in healthy and clinical tick bite skin biopsies. Dashed lines separate epidermis from dermis. In A–D, each dot represents one patient, and dotted lines connect intraindividual samples (n=4). Statistical significance was calculated using paired Student's *t* test (two tailed, *P < 0.05).

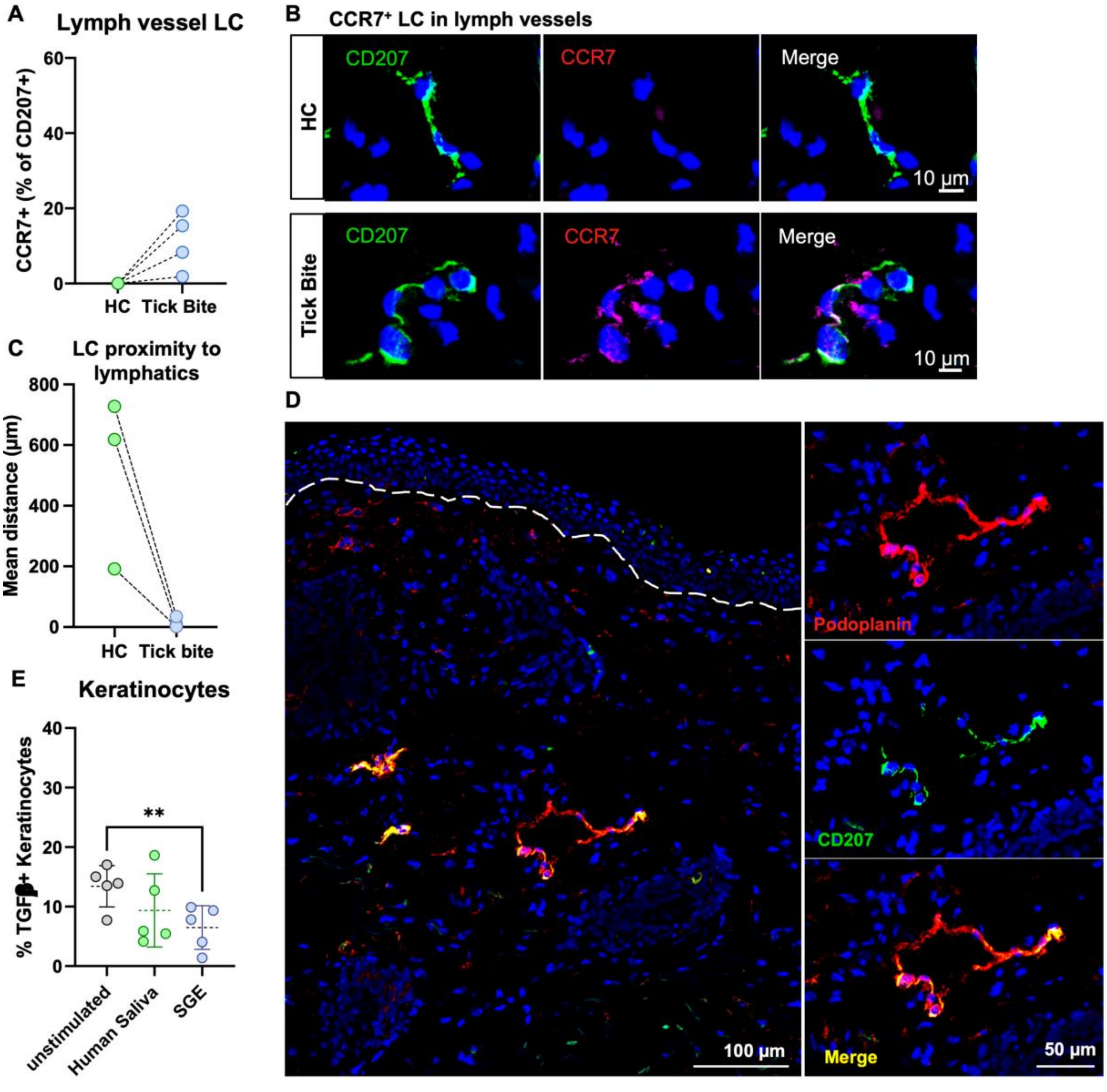

Supplementary Figure 3. LCs migrate to the dermis and are found in vicinity of lymph vessels after clinical tick bite. (A-B) Quantification (A) and representative IF images (B) of CCR7 (red) and CD207 (green) co-staining within a lymph vessel (n=5). (C) Mean distances (µm) of CD207+ LCs, defined as top 200 brightest CD207⁺ cells per sample from (D), to lymph vessel structures, defined as podoplanin⁺ cell clusters, in HC and clinical tick bites. (D)

Representative IF image of CD207 and podoplanin staining in clinical tick bite. (E) Flow cytometric analysis of TGF-β expression in keratinocytes after stimulation with human saliva or SGE (n=5, mean +/- SD). Statistical significance was calculated using paired Student's *t* test (two tailed) (A,B) and RM one-way ANOVA with Geisser-Greenhouse correction and Dunnett multiple comparison correction (*P < 0.05, **P < 0.01).

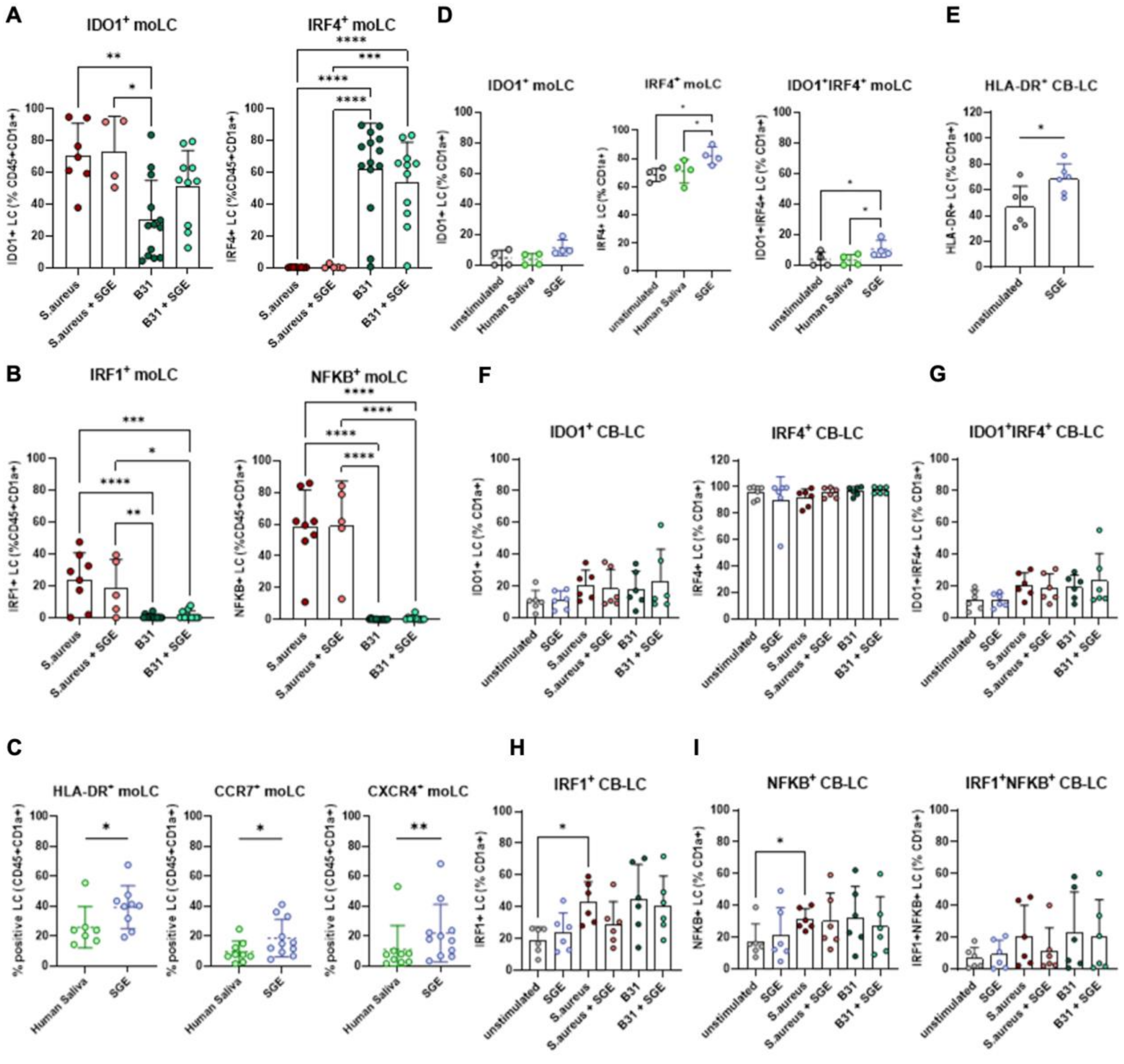

Supplementary Figure 4. LCs up-regulate tolerogenic TFs after stimulation with SGE and tick-borne *B. burgdorferi*. (A-B) Percentage of tolerogenic (IDO1, IRF4) (A) and immunogenic (IRF1, NFKB) (B) TF expressing moLCs, as analyzed by flow cytometry. (C) Expression of activation (HLA-DR) (n=7 for human saliva, n=9 for SGE) and migration (CCR7, CXCR4) (n=9) factors on moLC stimulated with either human saliva or SGE overnight. (D)

Percentages of IDO1+ and IRF4+ expression on moLC treated with human saliva or SGE (n=4). (E) Expression of HLA-DR as sign of activation with and without SGE stimulation in CB-LCs (n=6). (F,-I) Percentages of IDO1+ and IRF4+ (F,G), IRF1+ and NFKB+ (H,I) CB-LCs stimulated with SGE and/or *S. aureus* and *B. burgdorferi* (n=6). Data are plotted as mean values ± SD. Statistical significance was calculated using unpaired or paired Student's *t* test (two-tailed) for the comparison of two groups and ordinary one-way ANOVA with Turkey multiple comparison correction for the comparison of more than two groups (*P < 0.05; **P < 0.01, ***P < 0.001; ****P < 0.0001).

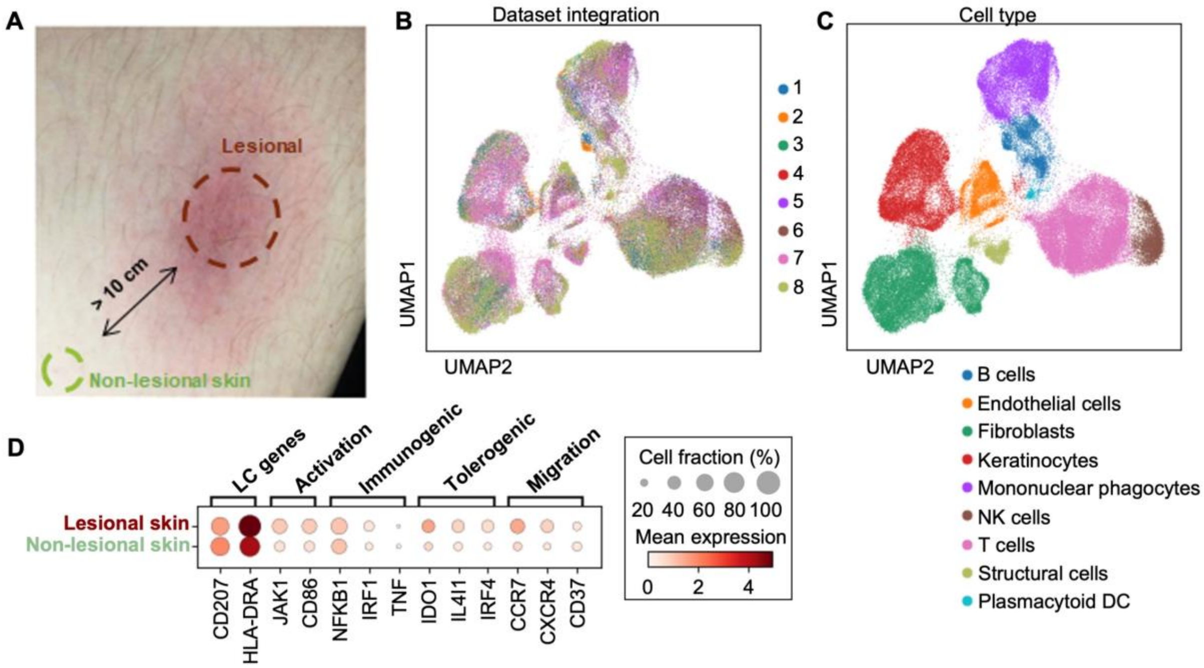

Supplementary Figure 5. Decreased numbers of LCs with a tolerogenic phenotype in a validation dataset of cutaneous Lyme disease lesions. (A) Representative clinical image and sampling strategy of an EM lesion. (B) UMAP projection of integrated datasets from Jiang *et al.* (samples 3-8) and newly generated (samples 1-2). (C) Cell type definition with canonical marker genes after Leiden clustering in cells from (B). (D) Dot plot showing the expression of genes associated with LC activation, migration and immunogenic and tolerogenic LC phenotypes in CD207-highly expressing cells from (C), within the mononuclear phagocytes cluster.

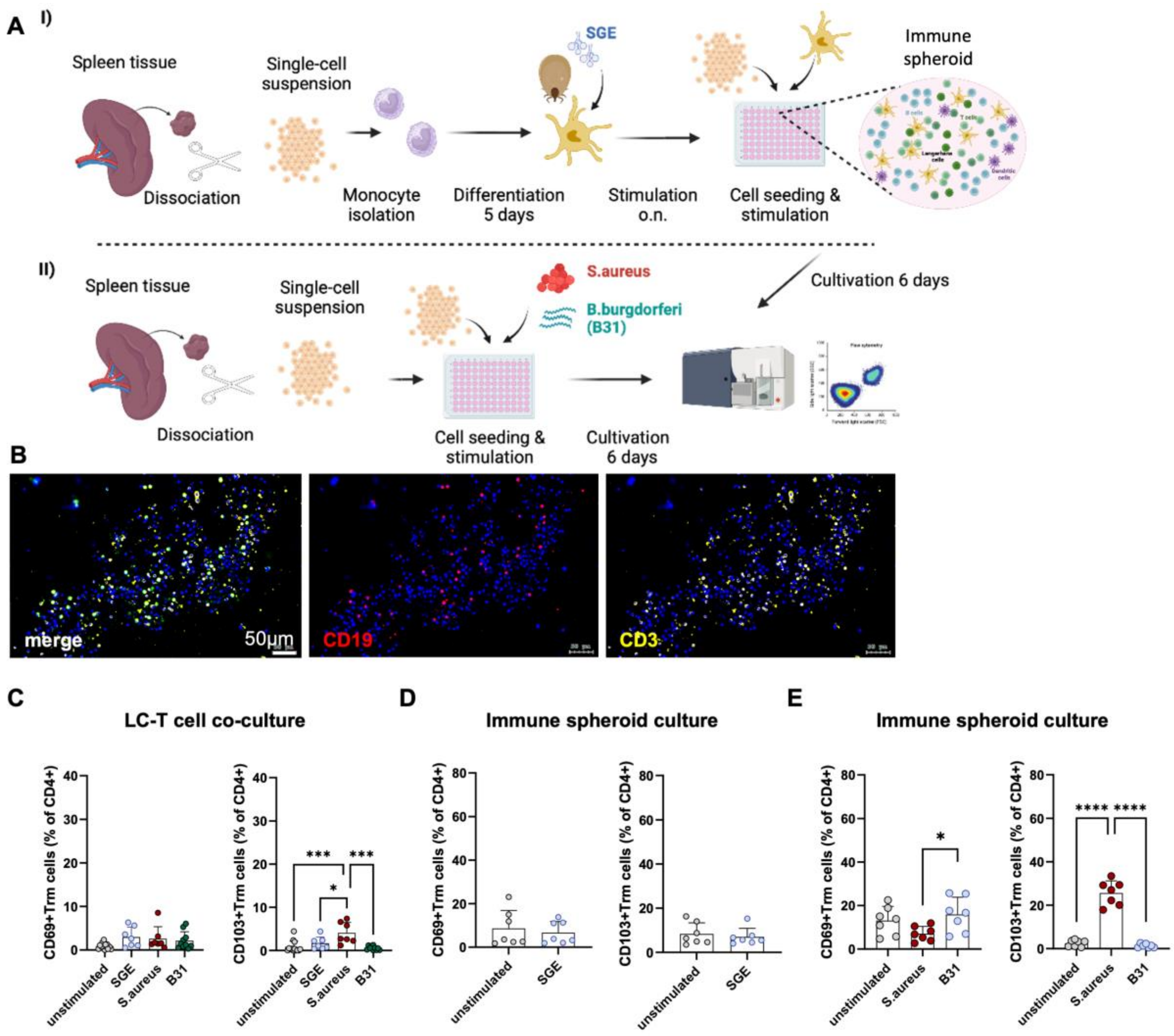

Supplementary Figure 6. T cell response in coculture and lymphoid tissue spheroid models. (A) Graphical representation of lymphoid tissue spheroid model using different settings: (I) Generation of single cell suspension, isolation of monocytes and differentiation into moLCs, with subsequent overnight SGE stimulation and co-cultivation with lymphoid tissue spheroids. (II) Generation of single cell suspension, seeding of lymphoid tissue spheroids and stimulation with pathogens. Created in BioRender. Stary, G. (2025) https://BioRender.com/4jpkqdg. (B) Visualization of immune spheroid model. Representative images of DAPI (blue), anti-CD19 (red), and anti-CD3 (yellow) immunofluorescence staining in an untreated immune spheroid model. (C-E) Flow cytometry data of LC-TC co-culture (C) and spheroid model (n= 6) (D,E) after co-incubation with stimulated moLCs (C,D) or direct bacteria stimulation (E). Data shown as mean percentages ± SD of CD69+ and CD103+ Trm among CD4+ T cells. Statistical significance was calculated using paired Student's *t* test (two-tailed) for the comparison of two groups and ordinary one-way ANOVA

with Turkey multiple comparison correction for the comparison of more than two groups (*P < 0.05; ***P < 0.001; ****P < 0.0001).

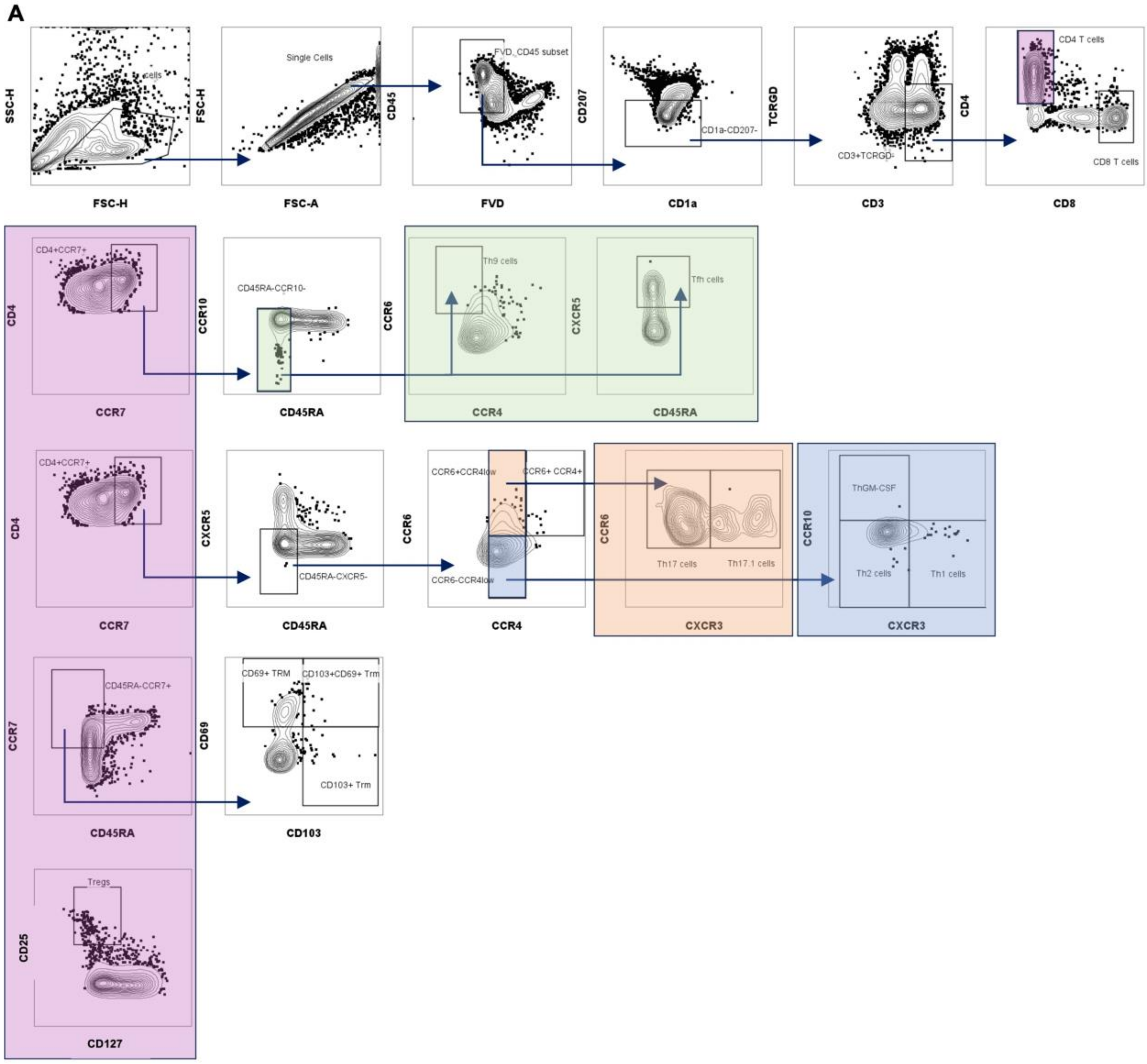

Supplementary Figure 7. Gating strategy for T cell subsets. Flow cytometry gating strategy to identify different T cell subsets in moLC-T cell co-cultures in the lymphoid tissue organoid model. Subset analysis was performed on live CD45+ cells.

Supplementary Tables

Sample No.	Sample type	Intraindividual HC	Lyme history	Borrelia-PCR
1	tick bite	yes	none	negative
2	tick bite	yes	none	negative
3	tick bite	yes	none	negative
4	tick bite	yes	none	negative
5	tick bite	yes	none	negative
6	tick bite	yes	none	negative
7	tick bite	yes	none	negative
8	Erythema migrans	yes	none	*B. afzelii*+
9	Erythema migrans	yes	none	*B. afzelii*+
				n=2

Supplementary Table 1. Clinical skin samples.

Antibody	Fluorophore	Clone	Source	Cat. No
CD1a	FITC	HI149	BD	#555803
CD207	PE	10E2	Biolegend	#352204
CCR7	AF647	A019D5	BD	#557734
CXCR4	PE	12G5	BD	#555974
Podoplanin	AF594	LpMab-21	Biolegend	#395006
AF488 anti-Oregon green /FITC	AF488	n/a	Life Technologies	#A11096
Goat anti-mouse IgG	AF549	n/a	Life Technologies	A11030

Supplementary Table 2. IF Antibodies. Primary and secondary antibodies used for IF staining of healthy and tick bite samples.

Antibody	Fluorophore	Clone	Source	Cat. No
moLC Differentiation/ Migration Panel				
HLA-DR	PE-Cy7	L234	Biolegend	#307616
CD45	PE-Dazzl	HI30	BD	#562279
CXCR4	PE	12G5	BD	#555974
CCR7	BV711	2D10	Biolegend	#353228
Viability Dye	eFluor780	n/a	Invitrogen	65-0865-14
e-cadherin	AF647	67A4	Biolegend	#324112
CD207	PerCP-Cy5.5	4C7	Biolegend	#144215
CD1a	FITC	HI149	BD	#555803
CB-LC Differentiation/ Migration				
e-cadherin	PE-Cy7	67A4	Biolegend	324116
CCR7	PE/Dazzle	G043H7	Biolegend	353236
CD1a	BV421	HI149	BD Biosciences	#563938
HLADR	BV510	G46-6	BD Biosciences	#563083
Viability Dye	efluor780	n/a	Invitrogen	65-0865-14
CXCR4	APC	12G5	Biolegend	306510
CD207	FITC	REA770	Miltenyi	#130-112-210
moLC Polarization Panel				
TNF-α	BUV395	Mab11	BD	#563996
Viability Dye	ZombieUV	n/a	Biolegend	#423107
IDO1	BUV737	eyedio	Invitrogen	#367-9477-42
CD11c	BV421	3.9	Biolegend	#301628
IRF4	eFluor450	3E4	Invitrogen	#48-9858-82
CCR7	BV510	G043H7	Biolegend	#353231
HLA-DR	BV570	L243	Biolegend	#307637

CD11b	BV785	ICRF44	Biolegend	#301345
CD1a	FITC	HI149	BD	#555803
CD207	PerCP-Cy5.5	4C7	Biolegend	#144215
CXCR4	PE	12G5	BD	#555974
CD45	PE-Dazzl	HI30	BD	#562279
IL-4	PE-Cy7	MP4-25D	Biolegend	#500824
IRF1	AF647	D5E4	Cell Signalling	#14105S
e-cadherin	AF647	67A4	Biolegend	#324112
pNFκB	AF750	polyclonal	Invitrogen	#BS-5512R-A750
CB-LC Polarization Panel				
IDO1	BUV737	eyedio	Invitrogen	#367-9477-42
CD1a	BV421	HI149	BD	#563938
HLA-DR	BV510	G46-6	BD	#563083
CD11b	BV711	ICRF44	Biolegend	#301344
IL-4	FITC	MP425D2	Biolegend	#500807
CXCR4	PerCP-Cy5.5	12G5	Biolegend	#306515
CD207	PE	DCGM4	Beckman Coulter	#PN IM3577
IRF4	PE-Cy7	IRF4.3E4	Biolegend	#646414
CCR7	PE-Dazzle	G043H7	Biolegend	#353236
IRF1	APC	DSE4	Cell Signaling	#14105S
IL-4	AF700	MP425D2	Biolegend	#500807
pNFκB	AF750	polyclonal	Invitrogen	#BS-5512R-A750
moLC-TC Polarization Panel				
CD45RA	BUV395	5H9	BD	#740315
Viability dye	Zombie UV	n/a	Biolegend	#423107
CD4	BUV563	OKT4	BD	750979
CD69	BUV661	FN50	BD	750213

CD8	BUV805	SK1	BD	612889
CD1a	BV480	HI194	BD	566147
CD3	BV510	SK7	Biolegend	344828
CD194/CCR4	BV605	L291H4	Biolegend	359418
CD25	BV711	2A3	BD	563159
CD196/CCR6	BV785	G034	Biolegend	353422
CD207	FITC	MB22-9F5	Miltenyi	130-098-349
CD45	PerCp	HI30	Biolegend	304026
TCRgd	PerCP-eFlour 710	B1.1	Invitrogen	46-9959-42
CD183/CXCR3	PE	1C6	BD	557185
CD197/CCR7	PE-CF594	150503	BD	562381
CD103	PE/Fire700	Ber-ACT8	Biolegend	350240
CD185/CXCR5	PE-Cy7	J252D4	Biolegend	356924
CCR10	APC	314305	R&D	FAB3478A
HLA-DR	APC-R700	G46-6	BD	565127
CD127	APC-eF780	RDR5	Invitrogen	47-1278-42
CD31	FITC	WM59	Biolegend	989002

Supplementary Table 3. Antibodies for flow cytometry. Primary labelled antibodies used for immunofluorescence stainings, flow cytometry staining in moLC and CB-LC differentiation, migration, polarization and moLC-T cell polarization experiments.

Supplementary video 1
Title: Cord blood-derived Langerhans cells
Description: [Live cell imaging of classical cord blood-derived Langerhans cell cultures without stimulation.]

Supplementary video 2
Title: Cord blood-derived Langerhans cells
Description: [Live cell imaging of cord blood-derived Langerhans cell cultures undergoing SGE stimulation.]
