# Supplementary material for: Human epidermal Langerhans cells induce tolerance and hamper T cell function upon tick-borne pathogen transmission
Source: Nat Commun. 2025 Nov 28;16:11715. doi: 10.1038/s41467-025-66821-6 (PMC12753675; doi:10.1038/s41467-025-66821-6)
Supplement: Supplementary file 2 — Description of Additional Supplementary Files [file 41467_2025_66821_MOESM2_ESM.pdf]

Title: Supplementary video 1

Description: Cord blood-derived Langerhans cells 86 Description: [Live cell imaging of classical cord blood-derived Langerhans cell cultures without 87 stimulation.]

Title: Supplementary video 2

Description: Cord blood-derived Langerhans cells 91 Description: [Live cell imaging of cord blood-derived Langerhans cell cultures undergoing SGE 92 stimulation.]
