# Supplementary material for: Human epidermal Langerhans cells induce tolerance and hamper T cell function upon tick-borne pathogen transmission
Source: Nat Commun. 2025 Nov 28;16:11715. doi: 10.1038/s41467-025-66821-6 (PMC12753675; doi:10.1038/s41467-025-66821-6)
Supplement: Supplementary file 5 — Reporting Summary [file 41467_2025_66821_MOESM5_ESM.pdf]

nature portfolio

Corresponding author(s): Georg Stary, Johanna Strobl

Last updated by author(s): May 28, 2025

Reporting Summary

Nature Portfolio wishes to improve the reproducibility of the work that we publish. This form provides structure for consistency and transparency in reporting. For further information on Nature Portfolio policies, see our Editorial Policies and the Editorial Policy Checklist.

Statistics

For all statistical analyses, confirm that the following items are present in the figure legend, table legend, main text, or Methods section.

n/a	Confirmed	
☐	☒	The exact sample size (n) for each experimental group/condition, given as a discrete number and unit of measurement
☐	☒	A statement on whether measurements were taken from distinct samples or whether the same sample was measured repeatedly
☐	☒	The statistical test(s) used AND whether they are one- or two-sided<br>*Only common tests should be described solely by name; describe more complex techniques in the Methods section.*
☒	☐	A description of all covariates tested
☒	☐	A description of any assumptions or corrections, such as tests of normality and adjustment for multiple comparisons
☐	☒	A full description of the statistical parameters including central tendency (e.g. means) or other basic estimates (e.g. regression coefficient) AND variation (e.g. standard deviation) or associated estimates of uncertainty (e.g. confidence intervals)
☐	☒	For null hypothesis testing, the test statistic (e.g. F, t, r) with confidence intervals, effect sizes, degrees of freedom and P value noted<br>*Give P values as exact values whenever suitable.*
☒	☐	For Bayesian analysis, information on the choice of priors and Markov chain Monte Carlo settings
☒	☐	For hierarchical and complex designs, identification of the appropriate level for tests and full reporting of outcomes
☒	☐	Estimates of effect sizes (e.g. Cohen's d, Pearson's r), indicating how they were calculated

Our web collection on statistics for biologists contains articles on many of the points above.

Software and code

Policy information about availability of computer code

Data collection	No software was used for data collection. Software used for data alignment, quantification, quality control and downsteam analysis of single cell datasets in this study are described in detail in manuscript 'Methods'.
Data analysis	Single-cell sequencing data were processed and analysed using publicly available software packages. Further information on analytical approaches used in the study are further detailed in manuscript 'Methods' section.<br>Software and packages versions used for computational analysis are as follows:<br>CellRanger Single Cell Software Suite 10x Genomics Inc (version 8.0.0 and 7.2.0); Reference genome (2020A reference genome, Chromium_Human_Transcriptome_Probe_Set_v1.0.1_GRCh38-2020-A, and refdata-cellranger-vdj-GRCh38-alts-ensembl-7.1.0); cellbender (version 0.2.1); Spaceranger (version 2.1.0); Scanpy (version 1.8.1 and 1.9.3); anndata (version 0.7.6); Scrublet (version 0.2.3); scvi-tools (version 0.20.3); GraphPad Prism (version 9);<br><br>Cell sorting decisions were executed by DIVA v.8 (BD Biosciences). FlowJo (v.10.6.2, BD Biosciences) was used for data analysis and presentation. Immunofluorescence microscopy images were analyzed in TissueQuest (version 6).

For manuscripts utilizing custom algorithms or software that are central to the research but not yet described in published literature, software must be made available to editors and reviewers. We strongly encourage code deposition in a community repository (e.g. GitHub). See the Nature Portfolio guidelines for submitting code & software for further information.

Data

Policy information about availability of data

All manuscripts must include a data availability statement. This statement should provide the following information, where applicable:

- Accession codes, unique identifiers, or web links for publicly available datasets
- A description of any restrictions on data availability
- For clinical datasets or third party data, please ensure that the statement adheres to our policy

scRNA-seq datasets related to this article are available via NCBI's Gene Expression Omnibus (GEO) database in form of de-identified count matrices, accession number GSE306532 (https://www.ncbi.nlm.nih.gov/geo/query/acc.cgi?acc=GSE306532).

Research involving human participants, their data, or biological material

Policy information about studies with human participants or human data. See also policy information about sex, gender (identity/presentation), and sexual orientation and race, ethnicity and racism.

Reporting on sex and gender	Data was obtained from donors of both sexes.
Reporting on race, ethnicity, or other socially relevant groupings	N/A
Population characteristics	N/A
Recruitment	Patients presenting with with tick bites or acute Lyme disease at the Department of Dermatology, Medical University of Vienna and the Department of Dermatology were asked to participate in the study. We did not detect recruitment biases in this study.
Ethics oversight	The study was approved by the local ethics committee at the Medical University of Vienna

Note that full information on the approval of the study protocol must also be provided in the manuscript.

Field-specific reporting

Please select the one below that is the best fit for your research. If you are not sure, read the appropriate sections before making your selection.

☒ Life sciences ☐ Behavioural & social sciences ☐ Ecological, evolutionary & environmental sciences

For a reference copy of the document with all sections, see nature.com/documents/nr-reporting-summary-flat.pdf

Life sciences study design

All studies must disclose on these points even when the disclosure is negative.

Sample size	As per the Human Cell Atlas white paper (https://www.humancellatlas.org/wp-content/uploads/2019/11/HCA_WhitePaper_18Oct2017-copyright.pdf), sample size was determined by recent experience using these technologies in relevant tissues. The sample size was made as large as it could be possible based on the availability of suitable materials.
Data exclusions	In this study, data exclusions were limited to removal of low quality single cells for downstream analysis of 'good quality' cells in scRNA-seq datasets. Any such data filtering steps are detailed in full in manuscript 'Methods'.
Replication	Biological as well as technical replicates were taken which reproduced the same results. IUnless stated otherwise in the figure legend, at least two technical replicates and at least 5 biological replicates were used.
Randomization	Randomization was not applicable in this study, because we describe an exploratory analysis in a discovery cohort.
Blinding	N/A: blinding is not applicable, because we did not perform a clinical study with specific clinical questions.

Reporting for specific materials, systems and methods

We require information from authors about some types of materials, experimental systems and methods used in many studies. Here, indicate whether each material, system or method listed is relevant to your study. If you are not sure if a list item applies to your research, read the appropriate section before selecting a response.

Materials & experimental systems

n/a	Involved in the study	
	x	Antibodies
x		Eukaryotic cell lines
x		Palaeontology and archaeology
	x	Animals and other organisms
x		Clinical data
x		Dual use research of concern
x		Plants

Methods

n/a	Involved in the study	
x		ChIP-seq
	x	Flow cytometry
x		MRI-based neuroimaging

Antibodies

Antibodies used

CCR10 – APC, Clone: 314305, Source: R\&D, Cat. No: FAB3478A
* **CCR7** – BV711, Clone: 2D10, Source: Biolegend, Cat. No: 353228
* **CCR7** – BV510, Clone: G043H7, Source: Biolegend, Cat. No: 353231
* **CCR7** – PE-Dazzle, Clone: G043H7, Source: Biolegend, Cat. No: 353236
* **CD1a** – BV421, Clone: HI149, Source: BD, Cat. No: 563938
* **CD1a** – BV480, Clone: HI194, Source: BD, Cat. No: 566147
* **CD1a** – FITC, Clone: HI149, Source: BD, Cat. No: 555803
* **CD103** – PE/Fire700, Clone: Ber-ACT8, Source: Biolegend, Cat. No: 350240
* **CD11b** – BV711, Clone: ICRF44, Source: Biolegend, Cat. No: 301344
* **CD11b** – BV785, Clone: ICRF44, Source: Biolegend, Cat. No: 301345
* **CD11c** – BV421, Clone: 3.9, Source: Biolegend, Cat. No: 301628
* **CD127** – APC-eFluor 780, Clone: RDR5, Source: Invitrogen, Cat. No: 47-1278-42
* **CD183/CXCR3** – PE, Clone: 1C6, Source: BD, Cat. No: 557185
* **CD185/CXCR5** – PE-Cy7, Clone: J252D4, Source: Biolegend, Cat. No: 356924
* **CD194/CCR4** – BV605, Clone: L291H4, Source: Biolegend, Cat. No: 359418
* **CD196/CCR6** – BV785, Clone: G034, Source: Biolegend, Cat. No: 353422
* **CD197/CCR7** – PE-CF594, Clone: 150503, Source: BD, Cat. No: 562381
* **CD207** – FITC, Clone: MB22-9F5, Source: Miltenyi, Cat. No: 130-098-349
* **CD207** – FITC, Clone: REA770, Source: Miltenyi, Cat. No: 130-112-210
* **CD207** – PE, Clone: DCGM4, Source: Beckman Coulter, Cat. No: PN IM3577
* **CD207** – PerCP-Cy5.5, Clone: 4C7, Source: Biolegend, Cat. No: 144215
* **CD25** – BV711, Clone: 2A3, Source: BD, Cat. No: 563159
* **CD3** – BV510, Clone: SK7, Source: Biolegend, Cat. No: 344828
* **CD4** – BUV563, Clone: OKT4, Source: BD, Cat. No: 750979
* **CD45** – PE-Dazzle, Clone: HI30, Source: BD, Cat. No: 562279
* **CD45** – PerCP, Clone: HI30, Source: Biolegend, Cat. No: 304026
* **CD45RA** – BUV395, Clone: 5H9, Source: BD, Cat. No: 740315
* **CD69** – BUV661, Clone: FN50, Source: BD, Cat. No: 750213
* **CD8** – BUV805, Clone: SK1, Source: BD, Cat. No: 612889
* **CXCR4** – APC, Clone: 12G5, Source: Biolegend, Cat. No: 306510
* **CXCR4** – PE, Clone: 12G5, Source: BD, Cat. No: 555974
* **CXCR4** – PerCP-Cy5.5, Clone: 12G5, Source: Biolegend, Cat. No: 306515
* **E-cadherin** – AF647, Clone: 67A4, Source: Biolegend, Cat. No: 324112
* **E-cadherin** – PE-Cy7, Clone: 67A4, Source: Biolegend, Cat. No: 324116
* **HLA-DR** – APC-R700, Clone: G46-6, Source: BD, Cat. No: 565127
* **HLA-DR** – BV510, Clone: G46-6, Source: BD, Cat. No: 563083
* **HLA-DR** – BV570, Clone: L243, Source: Biolegend, Cat. No: 307637
* **HLA-DR** – PE-Cy7, Clone: L234, Source: Biolegend, Cat. No: 307616
* **IDO1** – BUV737, Clone: eyedio, Source: Invitrogen, Cat. No: 367-9477-42
* **IL-4** – AF700, Clone: MP425D2, Source: Biolegend, Cat. No: 500807
* **IL-4** – FITC, Clone: MP425D2, Source: Biolegend, Cat. No: 500807
* **IL-4** – PE-Cy7, Clone: MP4-25D, Source: Biolegend, Cat. No: 500824
* **IRF1** – AF647, Clone: D5E4, Source: Cell Signaling, Cat. No: 14105S
* **IRF1** – APC, Clone: DSE4, Source: Cell Signaling, Cat. No: 14105S
* **IRF4** – eFluor450, Clone: 3E4, Source: Invitrogen, Cat. No: 48-9858-82
* **IRF4** – PE-Cy7, Clone: IRF4.3E4, Source: Biolegend, Cat. No: 646414
* **pNFκB** – AF750, Clone: polyclonal, Source: Invitrogen, Cat. No: BS-5512R-A750
* **TCRγδ** – PerCP-eFluor 710, Clone: B1.1, Source: Invitrogen, Cat. No: 46-9959-42
* **TNF-α** – BUV395, Clone: Mab11, Source: BD, Cat. No: 563996
* **Viability Dye** – eFluor780, Source: Invitrogen, Cat. No: 65-0865-14
* **Viability Dye** – ZombieUV, Source: Biolegend, Cat. No: 423107

Validation	All antibodies were validated in-house on human healthy tissue for IF prior to experiments.

Animals and other research organisms

Policy information about studies involving animals; ARRIVE guidelines recommended for reporting animal research, and Sex and Gender in Research

Laboratory animals	Frozen saliva from Ixodes ricinus ticks was acquired externally. No animals were used in the study.
Wild animals	NA
Reporting on sex	NA
Field-collected samples	NA
Ethics oversight	NA

Note that full information on the approval of the study protocol must also be provided in the manuscript.

Plants

Seed stocks	*Report on the source of all seed stocks or other plant material used. If applicable, state the seed stock centre and catalogue number. If plant specimens were collected from the field, describe the collection location, date and sampling procedures.*
Novel plant genotypes	*Describe the methods by which all novel plant genotypes were produced. This includes those generated by transgenic approaches, gene editing, chemical/radiation-based mutagenesis and hybridization. For transgenic lines, describe the transformation method, the number of independent lines analyzed and the generation upon which experiments were performed. For gene-edited lines, describe the editor used, the endogenous sequence targeted for editing, the targeting guide RNA sequence (if applicable) and how the editor was applied.*
Authentication	*Describe any authentication procedures for each seed stock used or novel genotype generated. Describe any experiments used to assess the effect of a mutation and, where applicable, how potential secondary effects (e.g. second site T-DNA insertions, mosiacism, off-target gene editing) were examined.*

Flow Cytometry

Plots

Confirm that:

- [x] The axis labels state the marker and fluorochrome used (e.g. CD4-FITC).
- [x] The axis scales are clearly visible. Include numbers along axes only for bottom left plot of group (a 'group' is an analysis of identical markers).
- [x] All plots are contour plots with outliers or pseudocolor plots.
- [x] A numerical value for number of cells or percentage (with statistics) is provided.

Methodology

Sample preparation	Single cell suspensions from skin punch biopsies were filtered and stained with antibody cocktails, as outlined in the "Methods".
Instrument	BDFAcsAria
Software	FACSDIva
Cell population abundance	Abundance of cell populations were determined post sorting by manually counting.
Gating strategy	As shown in the Suppl. Figure 7, for all FACS experiments, cells were gated based on FSC/SSC, live and single cells (FSC-H/FSC-A/SSC-W). For scRNA-seq, cells were taken from CD45+ positive gates.

- [x] Tick this box to confirm that a figure exemplifying the gating strategy is provided in the Supplementary Information.
